# Assessing the value of screening tools: reviewing the challenges and opportunities of cost-effectiveness analysis

**DOI:** 10.1186/s40985-018-0093-8

**Published:** 2018-07-13

**Authors:** Nicolas Iragorri, Eldon Spackman

**Affiliations:** 1 Department of Community Health Sciences and O’Brien Institute for Public Health, University of Calgary, Teaching, Research and Wellness Building, 3280 Hospital Drive NW, Calgary, AB T2N 4Z6 Canada; 2 Health Technology Assessment Unit, University of Calgary, Teaching, Research and Wellness Building, 3280 Hospital Drive NW, Calgary, AB T2N 4Z6 Canada

**Keywords:** Screening, Cost-effectiveness analysis, Value, Pre-symptomatic disease

## Abstract

**Background:**

Screening is an important part of preventive medicine. Ideally, screening tools identify patients early enough to provide treatment and avoid or reduce symptoms and other consequences, improving health outcomes of the population at a reasonable cost. Cost-effectiveness analyses combine the expected benefits and costs of interventions and can be used to assess the value of screening tools.

**Objective:**

This review seeks to evaluate the latest cost-effectiveness analyses on screening tools to identify the current challenges encountered and potential methods to overcome them.

**Methods:**

A systematic literature search of EMBASE and MEDLINE identified cost-effectiveness analyses of screening tools published in 2017. Data extracted included the population, disease, screening tools, comparators, perspective, time horizon, discounting, and outcomes. Challenges and methodological suggestions were narratively synthesized.

**Results:**

Four key categories were identified: screening pathways, pre-symptomatic disease, treatment outcomes, and non-health benefits. Not all studies included treatment outcomes; 15 studies (22%) did not include treatment following diagnosis. Quality-adjusted life years were used by 35 (51.4%) as the main outcome. Studies that undertook a societal perspective did not report non-health benefits and costs consistently. Two important challenges identified were (i) estimating the sojourn time, i.e., the time between when a patient can be identified by screening tests and when they would have been identified due to symptoms, and (ii) estimating the treatment effect and progression rates of patients identified early.

**Conclusions:**

To capture all important costs and outcomes of a screening tool, screening pathways should be modeled including patient treatment. Also, false positive and false negative patients are likely to have important costs and consequences and should be included in the analysis. As these patients are difficult to identify in regular data sources, common treatment patterns should be used to determine how these patients are likely to be treated. It is important that assumptions are clearly indicated and that the consequences of these assumptions are tested in sensitivity analyses, particularly the assumptions of independence of consecutive tests and the level of patient and provider compliance to guidelines and sojourn times. As data is rarely available regarding the progression of undiagnosed patients, extrapolation from diagnosed patients may be necessary.

## Background

Screening represents a cornerstone of preventive medicine. Its rationale is to identify disease during an early and pre-symptomatic stage [1]. With appropriate treatment, screening can result in disease prevention for those patients identified as at-risk. Early disease may be easier and less expensive to treat, which positions screening strategies as potentially sound investments for healthcare systems. Several countries have developed national screening programs that have led to increased disease detection rates and prevention [2, 3].

However, screening is not entirely risk-free and usually represents an immediate economic burden for systems with tight budget constraints. Some screening tools are associated with direct health risks (X-rays and radiation), and others might not provide a real additional value if, for instance, no follow-up treatment is available [1]. Additionally, tests need to be sufficiently reliable and accurate, since high proportions of false negatives or false positives might represent worse health outcomes and unnecessary diagnostic costs [4, 5]. To maximize value, an economic evaluation is a useful tool to compare the potential benefits, risks, and costs of different strategies and to inform resource allocation decisions. All health systems have scarce resources and are faced with opportunity costs; this means that any investment in a screening tool will come at the cost of other health services to the detriment of those patients who would have been treated [6].

Recognizing opportunity costs, healthcare systems may require that health interventions are both clinically and cost-effective to be considered for implementation [7]. Cost-effectiveness analysis (CEA) can be trial-based evaluations that use trial data to compare alternatives [8]; however, they are expensive to conduct and often require large sample sizes to obtain sufficient statistical power [9]. To overcome these challenges, model-based economic evaluations of screening tools have become a commonplace. Inputs are obtained from the best available sources and combined in mathematical models that replicate patient use of different strategies and provide a summary of costs and consequences for further analysis and comparison [10]. However, given that screening tools are used early in the treatment pathway, economic evaluations of screening strategies have many specific challenges to overcome. The objective of this study is to provide an overview of the different types of challenges and methodologies reported in the most recent cost-effectiveness analyses of screening strategies.

## Methods

### Eligibility criteria

A systematic review was conducted to identify the latest cost-effectiveness analyses (CEAs) of screening tools. Review and reporting followed the PRISMA (Preferred Reporting Items for Systematic Reviews and Meta-Analyses) guidelines [11]. Only research articles published in English and in 2017 were eligible for inclusion. CEAs comparing screening strategies versus no screening or other alternatives were included. There were no exclusion criteria based on the disease area. However, studies focusing on genomic screening and screening for blood transfusion, cost-benefit and cost-minimization studies, and review articles, editorial letters, news, study protocols, case reports, posters, and conference abstracts were excluded.

### Searches and study selection

We searched the online databases of EMBASE and MEDLINE. Search terms included Medical Subject Headings (MeSH), Emtree, and keywords for “mass screening” or screening, economic evaluation, and cost-effectiveness analysis. The last search was run on August 17, 2017. The search strategies can be found in Appendix 1 and Appendix 2. Two independent authors (NI and ES) screened all titles and abstracts. Any reference included by either reviewers at this stage was included for full-text review. This section was conducted independently and in duplicate. Disagreements at this stage were settled by discussion until a consensus was reached by both authors (NI and ES).

### Data extraction

We extracted the study characteristics and findings including the population, disease/condition, screening tools (strategies), comparators, perspective, time horizon, discounting, outcome or effectiveness measures (i.e., expected life years, quality-adjusted life years, cases detected), and incremental cost-effectiveness ratios (ICERs). A description of the findings was portrayed in a narrative synthesis. Results were compared to an economic evaluation focused on the early diagnosis and treatment of psoriatic arthritis (PsA) that is currently being developed by the authors (NI and ES).

## Results

**Fig. 1:**
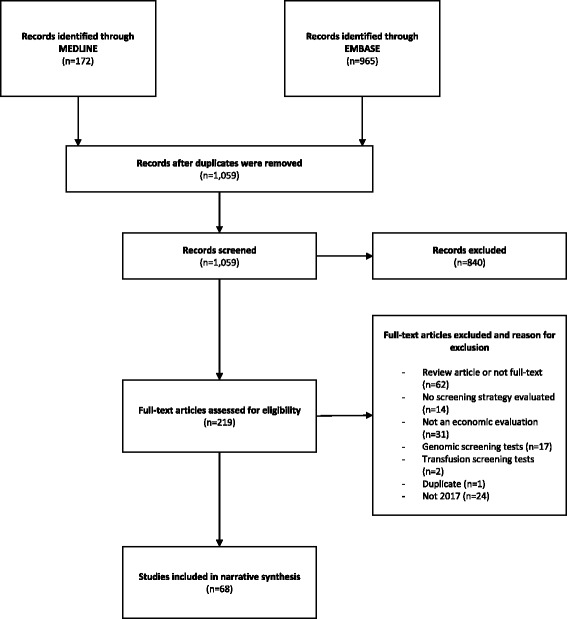
PRISMA flowchart. The PRISMA flow diagram details the search and study inclusion/exclusion process. It is a graphical representation of the flow of citations throughout the review

A total of 1059 records were found after 109 duplicates were removed. Two hundred nineteen articles were included for full-text assessment after 840 were excluded during the abstract screening stage (Fig. 1). Finally, 68 economic evaluations of screening tools were narratively synthesized (Table 1). A total of 26 studies (38.2%) evaluated the screening tools for cancer, 6 (8.8%) for hepatic disease, 5 (7.3%) for sexually transmitted disease, and 4 (5.8%) for heart disease. Twenty-nine (42.6%) added a “no screening” alternative for comparison. Thirty-five (51.4%) used quality-adjusted life years (QALYs) as the main outcome. Fifty-three studies (77.9%) modeled treatment options that followed screening and diagnostic testing. Finally, 7 studies (10.3%) concluded that the screening tool(s) they were evaluating were not cost-effective compared to current practice. The rest concluded that the implementation of screening tools had a high probability of being cost-effective. However, some specific recommendations regarding target populations, cost-effectiveness thresholds, and screening frequencies were made by some CEAs. Reported challenges and limitations of the economic evaluations were divided into three categories. The first one pertains to the screening pathway. It takes into account the test availability and sequencing, treatment options, accuracy, and patient compliance. The second describes the pre-symptomatic disease, prevalence, progression, and treatment effects. Finally, challenges with non-health benefits and spillovers are reported.

**Table 1:** Study characteristics

Authors	Country	Disease	Screening tools (strategies)	Comparator	Population	Time horizon	Perspective	Discounting	Monetary units	Effectiveness outcome	ICER	Conclusion of base case	Funding	Treatment
Albright et al. [55]	USA	Group B Streptococci	Universal screening with rectovaginal swab	No screening	Women with a prior cesarean delivery and a current singleton pregnancy planning to undergo a repeat cesarean	Lifetime	Healthcare	3%	2015 USD	Neonatal QALYs	Yes	Not CE	NA	Yes
Aronsson et al. [12]	Sweden	Colorectal cancer (CRC)	1. Fecal immunochemical test (FIT) twice 2. Colonoscopy (once) 3. FIT every 2 years 4. Colonoscopy every 10 years	No Screening	60-year-old Swedish	Lifetime	Healthcare	3%	EUR (no year)	QALYs	No	All strategies were CE vs no screening	SCREESCO study	Yes
Atkin et al. [18]	UK	Colorectal cancer	13 different Sttrategies	Each other and “no colonoscopy”	Individuals with intermediate-grade adenomatous polyps	Lifetime	NHS	4%	2012–2013 GBP	QALYs, ELYs	Yes	3-yearly ongoing colonoscopic surveillance without an age cut-off is CE	NIHR	Yes
Baggaley et al. [75]	UK	HIV	INSTI HIV1/HIV2 rapid antibody test	Not clear	Hackney Borough	40 years	NHS	4%	2012 GBP	QALYs	Yes	Screening is CE	NHS, NIHR	Yes
Barzi et al. [19]	USA	Colorectal cancer	13 screening tools: fecal occult blood test, Flex sig, colonoscopy, CT, DNA.	No screening	US population	35 years	Societal	3%	USD (no year)	Life years gained	No	Colonoscopy is CE	National Cancer Institute Core	Yes
Bleijenberg et al. [13]	Netherlands	Frailty	1. Electronic frailty screening instrument (EFSI) 2. EFSI and nurse-led care program	Usual care	Patients aged 60 or older	1 year	Societal	0%	2012 EUR	QALYs	No	EFSI has high probability of being CE. The combination showed less value for money.	NA	Yes
Cadier et al. [66]	France and USA	Hepatocellular Carcinoma	Biannual ultrasound + MRI + CT + biopsy	Real life	Patients with diagnosis of compensated cirrhosis	10 years	Healthcare	4%	2015 (Unknown)	Life years gained	Yes	Biannual ultrasound (gold standard) screening is CE	No funding	Yes
Wrenn et al. [79]	USA	Incidental gallbladder carcinoma	Cholecystectomy	Not clear	Cholecystectomies performed between 06/2009 and 06/2014	NA	NA	NA	NA	ELYs	No	Selective screening based on risk factors of specimen may be a more CE approach.	University of Vermont Medical Center Department of Surgery	Yes
Campos et al. [20]	50 low- and middle-income countries	Cervical cancer	1. Two-dose human papilloma virus (HPV) vaccination 2. One-time screening + treatment when neededz3. Cervical cancer treatment	Each other	1. 10-year-old girls2. 35-year-old women3. Women with cervical cancer	Lifetime	Payer	3%	2013 USD	DALYs	No	Both HPV vaccination and screening would be very CE	American Cancer Society	Yes
Chen et al. [45]	China	Hearing loss	Neonatal hearing screening	None	Newborns	15 and 82 years	NA	3%	2012 RMB	2012 RMB	No	Newborn hearing screening and intervention program in Shanghai is justified in terms of the resource input	National Natural Science Foundation of China	Yes
Cheng et al. [76]	China	Hepatitis E	1. Screening (HEV antibody) and vaccination 2. Universal vaccination	No vaccination	60-year-old cohort	16 years	Societal	3%	2016 USD	QALYs	Yes	Screening and vaccination is the most CE hepatitis E intervention strategy	Chinese National Natural Fund	Yes
Chevalier et al. [70]	France	Coronary artery disease	Maximal exercise test (ET)	None	Men aged > 35 years, with more than 2 h a week of training	NA	NA	NA	EUR (no year)	Cardiovascular disease cases	No	ET should be targeted at men with at least two cardiovascular risk factors	None	No
Chowers et al. [21]	Israel	Human immunodeficiency virus (HIV)	Prenatal HIV screening	Current policy	Newborns	100 years	Payer	4%	NIS (no year)	QALYs	No	Universal prenatal HIV screening is projected to be cost saving in Israel	NA	Yes
Coyle et al. [22]	Canada	Cancer	Computed tomography (CT) scan + occult cancer screening	Cancer screening alone	Patients with unprovoked VTE	12 months	Healthcare	0%	CAD (no year)	QALYs and Missed cancer case	No	CT scan of the abdomen/pelvis for the screening of occult cancer is not CE	Heart and Stroke Foundation of Canada	No
Cressman et al. [56]	Canada	Lung cancer	Low-dose computed tomography (LDCT)	Chest radiography	60-year-olds	30 years	Societal	3%	2015 CAD	QALYs	Yes	High-risk lung cancer screening with LDCT is likely to be considered CE	Terry Fox Research Institute	Yes
Crowson et al. [23]	USA	Vestibular schwannomas	Non-contrast screening Magnetic resonance imaging (MRI)	Full MRI protocol with contrast	Patients with asymmetric sensorineural hearing loss	NA	3rd-party payer	NA	USD (no year)	Useful results (True positives and true negatives)	No	A screening MRI protocol is more CE than a full MRI with contrast	None	No
Devine et al. [24]	Thailand-Myanmar	Perinatal hepatitis B	1. Hepatitis immunoglobulin (HBIG) after rapid diagnostic tests 2. HBIG after confirmatory test	Vaccination alone	Refugee and migrant population on the Thailand-Myanmar border	From first contact to childbirth	Healthcare	NA	USD (no year)	Perinatal infection of Hepatitis B	Yes	HBIG following rapid diagnostic test is CE	Wellcome-Trust Major Overseas Programme in SE Asia	No
Devine et al. [46]	Thailand-Myanmar	*Plasmodium vivax*	G6PD testing	[1] chloroquine alone [2] primaquine without screeningz	Refugee and migrant population on the Thailand-Myanmar border	1 year	Healthcare	NA	2014 USD	DALYs	Yes	G6PD RDTs to identify patients with G6PD deficiency before supervised primaquine is likely to provide significant health benefits	Welcome-Trust Major Overseas Programme in SE Asia	Yes
Ditkowsky et al. [25]	USA	Chlamydia trachomatis	Chlamydia screening	No Screening	Pregnant women aged 15–24	1 year	Healthcare	NA	2015 USD	2015 USD	No	Prenatal screening for C. trachomatis resulted in increased expenditure, with a significant reduction in morbidity to woman-infant pairs	None	Yes
Ethgen et al. [14]	France	Hepatitis C (HCV)	1. IFN + RBV + PI for F2–F4 2. IFN-based DAAs for F2–F4 3. All-oral, IFN-free DAAs for F2–F4 4. All-oral, IFN-free DAAs for F0–F4	No intervention	French baby-boomer population (1945–1965 birth cohorts)	20 years	Healthcare	4%	EUR (no year)	QALYs, liver-related deaths	No	HCV screening and access to all-oral DAAs is CE	AbbVie	Yes
Ferguson et al. [69]	Canada	Chronic kidney disease (CKD)	CKD screening	Usual care	Rural Canadian indigenous populations	45 years	Healthcare	5%	2013 CAD	QALYs	Yes	Targeted screening and treatment for CKD is CE	University of Manitoba, CIHR	Yes
Ferrandiz et al. [26]	Spain	Skin cancer	Clinical teleconsultations (CTC)	CTC + dermoscopic teleconsultation	Patients visiting 5 participating primary care centers because of concern over lesions suggestive of skin cancer	NA	NA	NA	EUR (no year)	Detected cases	No	Dermoscopic images improve the results of an internet-based skin cancer screening system	Health Council of the Regional Government of Andalusia-Spain	No
Goede et al. [27]	Canada	Colorectal cancer (CRC)	Fecal immunochemical testing (FIT)	Guaiac fecal occult blood testing and no screening	40-year-old screening participants at average risk of CRC	Varied (20 to 45 years)	Healthcare	3%	2013 CAD	QALYs	Yes	FIT was the most CE strategy	Ontario Ministry of Health and Long-Term Care	Yes
Gray et al. [47]	UK	Breast cancer	1. Risk 1 2. Risk 2 3. Masking 4. Risk 1 + masking	No screening	Women eligible for a National Breast Screening Program (NBSP)	Lifetime	NHS	4%	2014 GBP	QALYs	Yes	Risk stratified NBSPs were relatively CE compared to the UK NBSP	FP7-HEALTH-2012-INNOVATION-1	Yes
Gupta et al. [28]	USA	Cystic lung disease	High-resolution computed tomographic (HRCT) imaging	no HRCT screening	Patients with Spontaneous Pneumothorax	NA	Societal	3%	2014 USD	QALYs	Yes	HRCT image screening is CE	None	Yes
Haukaas et al. [44]	Norway	Tuberculosis (TB)	1. TST + IGRA 2. IGRA 3. IGRA for risk	No screening	Immigrants under 35 years of age from countries with a high incidence of TB	10 years	Healthcare	4%	2013 EUR	Avoided TB cases	Yes	IGRA is the optimal algorithm at a threshold above €28,400	None	No
Heidari et al. [29]	Iran	Hearing loss	1. AABR 2. OAE	Each other	Newborns	1 year	Healthcare	NA	IRR (no year)	Detected cases	No	AABR is the CE alternative compared to OAE	I.R. Iran’s National Institute of Health Research	No
Horn et al. [30]	USA	Substance abuse	1. Minimal screening 2. Screening, assessment and referral 3. 2 + brief intervention and follow-up	Each other	Patients from emergency departments of 6 clinical sites across the US	1 year	NA	NA	2013 USD	2013 USD	No	Resources could be better utilized supporting other health interventions.	NA	Yes
Htet et al. [71]	Myanmar	Pulmonary tuberculosis	Interventional model	Conventional model	Household contacts	5 months	NA	NA	USD (no year)	Detected cases	Yes	The interventional model was more CE than the modified conventional model.	NA	No
Hunter et al. [31]	USA	Breast cancer	Digital breast tomosynthesis	Full-field digital mammography	Patients undergoing screening mammography	1 year	NA	NA	2014 USD	Cancer detected	No	DBT is a cost-equivalent or potentially CE alternative to FFDM	NA	No
John et al. [48]	India	Glaucoma	Community screening	No screening	people aged 40–69 years in urban areas in India	10 years	Healthcare	3%	2015 INR	Additional treated cases, QALYs	Yes	A community screening program is likely to be CE	NZAID Commonwealth Scholarship	Yes
Keller et al. [68]	Australia	Prostate cancer	Serum prostate specific antigen (PSA) test every 2 years	Opportunistic screening	Australian male cohort aged between 50 and 69 years.	20 years	Healthcare	5%	2015 AUD	QALYs	Yes	PSA-based screening is not CE	University of Queensland	Yes
Kievit et al. [32]	Netherlands	Cardiovascular (CV) disease	CV risk profiling	No screening	Patients with rheumatoid arthritis (RA)	10 years	Medical	4% for costs and 1.5% for outcomes	EUR (no year)	QALYs	No	Screening for CV events in RA patients was estimated to be CE	NA	Yes
Kim et al. [49]	South Korea	Hepatitis C	One-time screening	No screening	People aged 40–70	5 years	Healthcare	5%	USD (no year)	QALYs	Yes	HCV screening and treatment is likely to be highly CE	Bristol-Myers Squibb Pharmaceuticals	Yes
Kim et al. [63]	USA	Human Papillomavirus	1. Cytology 2. HPV test 3. Co-test	Each other	US women	10–44 years	Societal	3%	USD (no year)	QALYs	No	Screening can be modified to start at later ages and at lower frequencies	National Cancer Institute of the National Institutes of Health	No
Lapointe-Shaw et al. [72]	USA	Carbapenemase-producing Enterobacteriaceae	Rectal swab screening	No screening	65-year-old patients admitted to a general medical inpatient service.	19.2 years	US Hospital	3%	2016 USD	QALYs	Yes	Screening inpatients for CPE carriage is likely CE	None	No
Lew et al. [58]	Australia	Colorectal cancer	Projected iFOBT screening	No screening	People aged 50–74	24 years	Health services	5%	2015 AUD	Life years gained	No	The program is highly CE	Cancer Institute NSW and Cancer Council NSW	Yes
Liow et al. [77]	USA	Bone malignancies	Routine femoral head histopathology	None	Patients that underwent primary total hip arthroplasty	4 years	NA	NA	2016 USD	QALYs	Yes	Routine femoral head histopathology may be CE	NA	Yes
Mo et al. [15]	China	Cervical cancer	1. Liquid-based cytology test + HPV DNA test 2. Pap smear cytology test + HPV DNA test 3. Visual inspection with acetic acid	No intervention	Adolescent girls (Above 12 years old)	Lifetime	Societal	3%	2015 USD	QALYs	Yes	The HPV4/9 vaccine with current screening strategies was highly CE	Japan Society for the Promotion of Sciences	Yes
Morton et al. [50]	UK	Breast cancer	Mammography	No screening	Females over 45 years old	20 years	NHS	4%	2016 GBP	QALYs	Yes	Calculations suggested that breast cancer screening is CE	NA	Yes
Mullie et al. [51]	Canada and USA	Latent tuberculosis	1. Tuberculin skin test 2. QuantiFERON®-TB-Gold In-Tube	Each other	Healthcare workers	20 years	Healthcare	3%	2015 CAD	QALYs	Yes	Annual tuberculosis screening appears poorly CE	McGill University, CIHR	Yes
Petry et al. [16]	Germany	Human papillomavirus	1. HPV test followed by Pap cytology 2. HPV test followed by cytology 3. HPV test followed by colposcopy 4. Co-testing with HPV and Pap	Pap cytology	Women aged 30–65	10 years	NA	3%	EUR (no year)	Avoided deaths	No	The greatest clinical impact was achieved with primary HPV screening (with genotyping) followed by colposcopy	Hoffmann-La Roche	Yes
Phisalprapa et al. [33]	Thailand	Nonalcoholic fatty liver disease	Ultrasonography screening	No screening	50-year-old metabolic syndrome patients	Lifetime	Societal	3%	2014 USD	QALYs	Yes	Ultrasonography screening for NAFLD with intensive weight reduction program is CE	NA	Yes
Pil et al. [59]	Belgium	Skin Cancer	Total body skin examination (TBSE)	Lesion-directed screening	Belgian population over 18 years of age	50 years	Societal	Outcomes at 1.5% and costs at 3%	EUR (no year)	QALYs	Yes	1-time TBSE is the most CE strategy	The LEO Foundation and the Belgian Federation Against Cancer	Yes
Prusa et al. [80]	Austria	Toxoplasmosis	Prenatal screening	No screening	Birth cohorts from 1992 to 2008 and	20 years	Societal	3%	2012 Euro	Life and productivity loss	No	Cost savings of prenatal screening for toxoplasmosis and treatment are outstanding	None	Yes
Requena-Mendez et al. [34]	All Europe	Chagas disease	*T. cruzi* serological screening	No screening	Latin American adults living in Europe	Lifetime	Healthcare	3%	EUR (no year)	QALYs	YES	Screening for Chagas disease in asymptomatic Latin American adults living in Europe is a CE strategy.	European Commission 7th Framework Program	Yes
Roberts et al. [60]	Australia	Rheumatic heart disease	Echocardiographic screening	Screening every other year and no screening	Indigenous Australian Children	40 years	Healthcare	5%	2013 AUD	DALYs, heart failure, surgery	Yes	Echocardiographic screening is CE assuming that RHD can be detected ≥ 2 years earlier by screening	University of Western Australia	Yes
Rodriguez-Perez et al. [64]	Spain	Type 2 diabetes	DIABSCORE	HbA1c or blood glucose	Adult primary care patients in Spain	NA	NA	NA	EUR (no year)	Cases detected	No	DIABSCORE is a CE and valid method for opportunistic screening of type 2 diabetes	Carlos III Health Institute	No
Saito et al. [35]	Japan	Gastric cancer	ABC method: HPA and measuring serum PG concentrations	Annual endoscopic screening	50-year-old Japanese individuals who have high gastric cancer incidence and mortality who had not undergone *H. pylori* eradication	30 years	Healthcare	2%	2014 USD	Lives saved and QALYs	Yes	ABC method cost less and saved more lives	Niigata University of Health and Welfare	Yes
Schiller-Fruehwirth et al. [36]	Austria	Breast cancer	1. Organized screening 2. Opportunistic screening	No screening	40-year-old asymptomatic women	Lifetime	Healthcare	3%	2012 EUR	Life years gained	Yes	The decision to adopt organized screening is likely an efficient use of limited health care resources in Austria	Main Association of Social Security Institutions	Yes
Selvapatt et al. [65]	UK	Hepatitis C	HCV testing	No screening	All persons attending a London DTU	Lifetime	Healthcare	4%	2013 GBP	ELYs, QALYs	Yes	Concludes cost effectiveness of outreach testing and treatment of hepatitis	Biomedical Research Council to Imperial College Department of Hepatology	Yes
Sharma et al. [61]	Lebanon	Cervical cancer	1. Cytology 2. HPV DNA screen	No screening	Women aged 25–65 years	NA	Societal	3%	I$ (no year)	Years of life saved	Yes	Increasing coverage to 50% with extended screening intervals provides greater health benefits	None	Yes
Smit et al. [73]	Belgium	Tuberculosis	X-ray screening	No screening	Risk groups: prisoners, youth in detention centers, undocumented migrants	1 year	Flemish Agency for Care and Health	0%	2013–14 EUR	Detected cases	No	Tuberculosis screening is relatively expensive	Flemish Agency for Care and Health	No
Ten Haaf et al. [52]	Canada	Lung cancer	Computer tomography	No screening	Persons born between 1940 and 1969	Lifetime	Healthcare	3%	2015 CAD	Life years gained, false positive screen	Yes	Lung cancer screening with stringent smoking eligibility criteria can be CE	Clinical Evaluative Sciences	Yes
Teng et al. [62]	New Zealand	*Helicobacter pylori* infection, gastric cancer	1. Fecal antigen 2. Serology	Current practice	Total population and targeted Māori (25–69 years old)	Lifetime	Healthcare	3%	2011 USD	QALYs	Yes	Screening was likely to be CE particularly for indigenous populations	Health Research Council of New Zealand	Yes
Tjalma et al. [37]	Belgium	Cervical cancer	Dual stain cytology	Cytology	Women between 25 and 65 years of age	60 years	Healthcare	NA	EUR (no year)	QALYs	Yes	Diagnostic cytology benefits all stakeholders involved in cervical cancer screening	NA	Yes
Tufail et al. [38]	UK	Diabetic retinopathy	Automated diabetic retinopathy image assessment systems (ARIAS)	Human graders	Patients with a diagnosis of diabetes mellitus who attended their annual visit at the diabetes eye-screening program	NA	NHS	4%	2013–2014 GBP	Appropriate screening outcome	No	ARIAS have the potential to reduce costs and to aid delivery of DR screening	Novartis	No
Meulen et al. [39]	Netherlands	Colorectal cancer (CRC)	1. Fecal immunology test 2. gFOBT 3. Sigmoidoscopy	Each other	Screening-naive subjects ages 50 to 74 years, living in the southwest of the Netherlands	Lifetime	Healthcare	3%	2012 EUR	Positivity rates, detection of adenoma and CRC, QALYs	Yes	Screening stratified by gender is not more CE than uniform FIT screening	NA	Yes
van Katwyk et al. [53]	Canada	Diabetic retinopathy	Extended coverage of diabetic eye examination	Usual care	Prince Edward Island residents over 45 years of age who had diabetes	30 years	Healthcare	5%	2015 CAD	QALYs	Yes	Extending public health coverage to eye examinations by optometrists is CE	CIHR	Yes
van Luijt et al. [67]	Norway	Breast cancer	Mammography	No screening	Norway female population	Lifetime	Societal	4%	2014 NOK	QALYs	No	The NBCSP is a highly CE measure to reduce breast cancer specific mortality	Research Council Norway	Yes
Wang et al. [17]	China	Chronic kidney disease	1. Day 1 2. Random 3. Day 1 + random 4. Day 1+ random + day 2	Each other	Outpatients admitted to Peking University First Hospital from January 2013 to January 2014	30 years	Societal	5%	CNY (no year)	QALYs	Yes	Combining two first morning urine samples and one randomized spot urine sample is CE	National Key Technology R&D Program of the Ministry of Science and Technology	Yes
Welton et al. [40]	England and Wales	Atrial fibrillation	1. Single systematic population screen 2. Single systematic opportunistic screen	No screening	General population in England and Wales	Lifetime	NHS	4%	2015 GBP	QALYs	Yes	Population-based screening is likely to be CE	NIHR	Yes
Whittington et al. [74]	USA	*Staphylococcus aureus* infection	1. Universal decolonization 2. Targeted decolonization 3. Screening and isolation	Each other	Hypothetical cohort of adults admitted to the Intensive care unit.	1 year	Hospital	NA	2015 USD	QALYs	Yes	This study supports updating the standard practice to a decolonization approach.	NA	No
Williams et al. [41]	USA	Prosthetic joint infection	1. 4 swabs decolonization 2. 2 swabs 3. Nasal swab alone	No screening and decolonization	Hip and knee replacement patients	NA	Societal	NA	2016 USD	Cases of prosthetic joint infections	No	The 2-swab and universal-decolonization strategy were most CE	None	Yes
Yang et al. [54]	Taiwan	Lung cancer	1. Computed tomography (CT) 2. Radiography	No screening	Smokers between 55 and 75 years of age	Lifetime	Healthcare	3%	2013 USD	QALYs	Yes	Low-dose CT screening for lung cancer among high-risk smokers would be CE in Taiwan	Ministry of Science and Technology, and the National Cheng Kung University Hospital	Yes
Yarnoff et al. [42]	USA	Chronic kidney disease (CKD)	CKD risk scores	No screening	US population	Lifetime	Healthcare	3%	2010 USD	QALYs	Yes	CKD risk scores may allow clinicians to cost-effectively identify a broader population for CKD screening	Centers for Disease Control and Prevention	Yes
Yoshimura et al. [78]	Japan	Osteoporosis	Screening and alendronate therapy	No screening and no therapy	Postmenopausal women over 60 years	5 years	Healthcare	3%	USD (no year)	QALYs	Yes	Screening and treatment would be CE for Japanese women over 60 years.	Ministry of Education, Culture, Sports, Science and Technology	Yes
Zimmermann et al. [43]	Kenya	Cervical cancer	1. Visual inspection with acetic acid (VIA) 2. Papanicolaou smear 3. Testing for human papillomavirus (HPV)	Cryotherapy without screening	Hypothetical cohort of 38-year-old women	Lifetime	Societal	3%	2014 USD	ELYs	No	VIA was most CE unless HPV could be reduced to a single visit	NA	Yes

*QALYs* quality-adjusted life years, *ELYs* expected life years, *RMB* Renminbi, *USD* United States dollar, *CAD* Canadian dollar, *AUD* Australian dollar, *EUR* euro; *GBP* British pound, *NIS* Israeli new shekel, *IRR* Iranian rial, *CNY* Chinese yuan, *INR* Indian rupee, *NOK* Norwegian krone, *CE* cost-effective, *NA* not applicable

### Screening pathway

The value of the screening test is dependent on the full screening pathway. This refers to the screening test and the subsequent follow-up undertaken because of the results of the screening. The review identified multiple studies that evaluated different screening pathways by modifying the order in which screening tests were administered [12–17]. This allowed investigators to determine trade-offs between potential screening sequences. However, these models are dependent on data availability, and lots of different types of evidence are necessary to inform the screening pathway including screening and diagnostic test accuracy and screening compliance. Most studies explored challenges such as conditional test accuracy, a lack of a diagnostic gold standard, outcomes of false positives and false negatives, or screening compliance.

#### Accuracy

Twenty-five studies (36.7%) explicitly reported challenges regarding screening test accuracy [18–43]. One common challenge was the lack of data on test accuracy. In some cases, authors had to assume the accuracy of the screening test [28, 30, 32, 33]; more commonly, it was assumed that tests had the same performance regardless of prior testing [19, 34]. This assumption is particularly important when different sequences of screening and diagnostics tests are being evaluated. Accuracy assumptions were often tested using different combinations of sensitivity and specificity. Barzi et al. modeled a hypothetical test and, through model iterations, determined the combination of test sensitivity and specificity that would yield optimal results in terms of cost-effectiveness [19]. Crowson et al. undertook a two-way sensitivity analysis of sensitivity and specificity to determine their importance to health outcomes and costs [23]. Sensitivity analyses are useful tools to evaluate the uncertainty around test accuracy estimates. These analyses allow a threshold to be determined at which a specific screening tool would result in a cost-effective strategy.

To understand the implications of screening on patients’ health, it is important to model the outcomes of any follow-up diagnostic tests. However, one common difficulty is that there is usually no information on the accuracy of the diagnostic test in the screen-positive population. A few assumptions were made to account for this uncertainty. A study in Thailand for non-alcoholic fatty liver disease used pooled estimates of diagnostic accuracy from a meta-analysis assuming independence between the screening and diagnostic accuracy [33]. Chowers et al. tested different accuracy rates for HIV diagnostic tests with sensitivity analyses [21]. Other studies assumed specific accuracy estimates (usually 100%) and acknowledged the limitations, such as potentially overestimating cost-effectiveness estimates by excluding pertinent costs associated to misclassified patients [22, 29, 44].

#### False positive and negative outcomes

Screening and diagnostic accuracy determines the proportion of patients who will continue to receive treatment or further follow-up. It is important to understand the health outcomes of all patients screened. Patients identified as false positive or false negative are particularly difficult to consider in cost-effectiveness analysis given the lack of data on these patients. Costs and outcomes for patients who followed incorrect screening and treatment pathways were included in 22 (32.3%) of the studies [12, 17, 18, 21, 23–25, 29, 36, 40, 42, 43, 45–54]. Even though some cost-effectiveness analyses identified false positives in the screening pathways, one alternative was to assume 100% accurate diagnostic tests; this meant patients identified incorrectly during screening would never go on to inappropriate treatment [29, 42, 49]. In these cases, there were extra diagnostic costs, but no treatment-specific costs or outcomes were pertinent. Health outcomes may be overestimated when assuming 100% accurate diagnostic tests. Alternatively, some studies assumed that diagnostic tests were not perfect and included costs and health consequences of the incorrect treatment of false positive patients, such as healthy patients receiving unnecessary treatment and having side effects [17, 43, 48, 53, 54]. Whenever a treatment poses a considerable threat to false positives (or a considerable monetary cost), CEAs should acknowledge and include these scenarios. When false negative patients were modeled, it was assumed that they would progress at the same rate as untreated patients and were usually identified as being sick once symptoms appear [17, 21, 45, 46, 48]. This is comparable to the pathway for all sick patients under a “no screening” arm. A high proportion of false negatives (i.e., tests with low sensitivity) will translate to fewer identified sick patients. Depending on the disease, tests, costs, and health outcomes, a CEA could evaluate whether repeated testing is worth implementing to reduce this proportion of patients. Four studies failed to model false positives and/or negatives after acknowledging their potential effect to the evaluation [12, 18, 25, 36].

#### Compliance

Screening pathways are greatly altered by different rates of participation and compliance. Screening is only effective if the target population and healthcare providers are engaged. Twenty-nine evaluations (42.6%) identified patient participation and compliance as an important model parameter [12, 14, 16–20, 25, 27, 28, 32, 36, 37, 43–46, 48–51, 55–62]. Morton et al. reported that the results of a national breast cancer screening program in the UK would be impacted by the proportion of the at-risk population who decided to participate [50]. Lower compliance translates to lower screening and diagnostic costs, but also represents a higher burden of disease if non-compliers are diagnosed at later and more expensive-to-treat stages of disease. Screening can also raise costs without improving health outcomes if identified patients fail to follow further recommended treatment due to unreliable testing. John et al. also modeled non-compliers who had a chance of getting sick and being identified by opportunistic screening [48]. Additionally, studies such as that conducted by Aronsson et al. explain how compliance rates are dependent on the screening tool to be evaluated [12, 19]. They model colonoscopy and fecal immunochemical tests (FIT) to screen for colorectal cancer, and take into account the different compliance rates for each alternative. Since colonoscopy is expected to make people more uncomfortable than the FIT, less people are expected to comply with the former [12, 19]. To test this, willingness-to-pay to avoid colonoscopy was estimated [12]. However, information about the compliance rates for different screening tests was rarely available. Ten studies (14.7%) assumed a 100% compliance rate [17, 25, 27, 28, 32, 45, 51, 55, 57, 59, 63]. The effect of this assumption over cost-effectiveness estimates depends on the specific evaluation being conducted, specifically the trade-off between lower screening costs and worse health outcomes due to unidentified disease.

### Pre-symptomatic disease

Disease prognosis and patient evolution from pre-symptomatic stages of disease were modeled in most cases to estimate aggregate costs and outcomes. All included studies but 2 (3%) [38, 64] explicitly commented on challenges encountered while trying to adequately model disease progression and patient transition through health states. Pre-symptomatic disease refers to the point in progression when the disease is developing but no symptoms are apparent. This is the point when screening tools are useful but usually when there is very little data about progression of the disease. Once identified as having a disease, more data is available for modeling cost-effectiveness.

#### Prevalence/incidence

Screening models often focus on at-risk populations. Incidence rates are used to determine the proportion of patients who enter the models at pre-symptomatic stages. This is useful for scenarios with repeated screening procedures, as a dynamic model can be developed to evaluate repeated screening processes while taking into account new at-risk patients [47]. On the other hand, some studies included population-specific incidence rates [19, 35, 47]. A different approach consists on evaluating one-time-only screening procedures targeting prevalent disease [49]. Deciding between repeated versus one-time testing depends on the type of disease and population of the evaluation. A one-time test for tuberculosis might be appropriate for immigrant populations, while testing for lung cancer among smokers is recommended to be carried out repeatedly. The sequence and frequency of tests can be tested through modeling to determine the cost-effective option. Sensitivity analyses determined that cost-effectiveness estimates were highly sensitive to changes in prevalence and incidence estimates [25, 49, 65]. Testing for a rare disease might not result cost-effective compared to a common disease given a similar health and economic burden.

#### Pre-symptomatic disease progression

Once an at-risk population is identified, some cost-effectiveness analyses focused on modeling the pre-symptomatic stages of disease. There is a time interval before clinical symptoms appear and after disease onset where disease is identifiable by screening tools. This timeframe, also called sojourn time, is a major challenge for CEA since progression of pre-symptomatic disease if often unknown (Table 2). Uncertainty around sojourn time was tested by 3 studies (4.4%) [36, 60, 66]. van Luijt et al. determined a fixed preclinical stage of breast cancer where disease could be identified by screening [67]. This study also allowed for disease regression or progression to more advanced pre-symptomatic stages. Atkin et al. modeled similar pre-symptomatic stages for colorectal cancer and adenoma [18]. Sensitivity analyses allowed to estimate the effect of varying the interval for sojourn time on cost-effectiveness. These studies concluded that longer sojourn time represented improved disease identification rates.

**Table 2:** Summary of methodological issues and suggestions to develop CEAs of screening tools

Issues	Suggestions
Screening/diagnostic test accuracy	Model iterations with two-way sensitivity analyses using different combinations of sensitivity and specificity to determine a threshold at which screening becomes cost-effective. Assuming 100% accuracy might overestimate cost-effectiveness estimates.
Modeling false positive and negative results	Building a pathway for false positives and false negatives that includes their costs and health outcomes. For false positives, it is important to include costs and health outcomes associated to unnecessary diagnostics and treatment. For false negatives, it is important to include the costs and health outcomes of a delayed diagnosis.
Compliance rates	Model the compliance rate of patients and healthcare delivery professionals. Compliance rates are particularly important when repeated screening is being recommended, since low compliance may mean that the costs of early testing are wasted if further testing is not done.
Prevalence/incidence	Screening programs are usually conducted repeatedly over time. Dynamic models (incidence based) can be developed to evaluate repeated screening processes while considering new at-risk patients. One-time-only screening procedures only take into account prevalent disease.
Pre-symptomatic progression rates	Population-specific progression rates are often difficult to find for pre-symptomatic disease. Extrapolation from the clinical phase, or from similar conditions, could represent a first step to tackle the uncertainty around these parameters. Sensitivity analyses should determine how progression rates are expected to affect cost-effectiveness estimates.
Sojourn time	Sojourn time determines when screening is appropriate. This is a crucial input into a screening model and there is rarely evidence to estimate it. Creating various scenarios with different sojourn times may allow the investigators to estimate its impact on cost-effectiveness estimates. Different sojourn times will affect the cost-effectiveness of different test frequencies and should be evaluated using cost-effectiveness modeling.
Treatment and health outcomes	CEAs of screening tools should always include follow-up diagnostic and treatment. Quality-adjusted life years are appropriate to account for health outcomes, but these should be specific to the population being evaluated. Every potential health outcome needs to be accounted for including side effects of screening and/or diagnostic tests.
Non-health-related spillovers	Evaluating a screening tool from a societal perspective requires the inclusion of all non-health costs and outcomes. It is important to understand the trade-offs between the different types of costs and benefits. The inclusion of non-health costs and outcomes has important distributional assumptions and will value patients differently.

Modeling patient progression during the sojourn time, i.e., through pre-symptomatic health states, remains a challenge. Three studies extrapolated progression rates from symptomatic disease stages to pre-symptomatic disease [18, 56, 68]. In some cases, fast progressing disease may cause death before diagnosis. Death rates for pre-symptomatic disease were available for colorectal cancer using Kaplan-Meier estimators from lifetime data [18], health state-specific mortality risks in chronic kidney disease [69], and gastric cancer [62]. Additionally, based on differential progression rates and life expectancy, two studies evaluated the potential effect of lead time bias in their studies [35, 54]. This bias explains how early diagnosed patients might not experience an increase in expected survival, but instead spend longer periods under treatment. This effect gives the illusion of higher survival expectancy [35], resulting in biased cost-effectiveness estimates. Survival has a major impact over health-related outcomes in CEAs, and assuming a higher rate will overestimate the health benefits. This is one example of a model input that is likely to affect the cost-effectiveness of a screening tool and should be tested in sensitivity analyses. Yang et al. used population matching (cancer cases vs general population) and a difference in difference methodology to determine if early diagnosis provided improved life expectancy [54]. Both studies showed differential survival rates favoring patients who were diagnosed early after accounting for potential lead time bias [35, 54].

### Treatment effect and health outcomes

According to the WHO, screening interventions are expected to provide treatment alternatives for those patients with identified cases of disease [1]. However, 15 studies (22%) failed to model a treatment pathway [22–24, 26, 29, 31, 38, 44, 57, 64, 70–74]. The main outcomes captured by these studies were the following: cases detected, missed cases, avoided cases, and identified true positives and true negatives. Decision trees were most commonly used for these modeling tasks. However, these models are insufficient for making reimbursement decisions, since efficacious interventions or therapies are required to follow screening and diagnostic procedures to improve patients’ health. Without these benefits, screening procedures are not capturing all consequences, leading to incomplete CEAs. On the other hand, studies that modeled treatment pathways captured different health outcomes to evaluate cost-effectiveness of screening strategies. Quality-adjusted life years (QALYs) were estimated by 39 studies (57.3%) [12–15, 17, 18, 21, 27, 28, 32–35, 37, 39, 40, 42, 45–51, 53–57, 59, 62, 65, 67–69, 72, 74–78], and expected life years (ELYs) by 10 (14.7%) [18, 19, 36, 43, 52, 58, 65, 66, 79, 80]. Utilities were widely used, and the following challenges and methodologies were reported: Chowers et al. acknowledged having underestimated QALY outcomes in their prenatal HIV screening evaluation by excluding maternal utility measures. Additionally, treatment for false positives and its repercussions were excluded, even though treatment for healthy newborns is expected to cause disutility [21]. Ferguson et al. observed there was a difficulty assigning utilities for patients with undiagnosed chronic kidney disease. Therefore, they assumed similar utilities for undiagnosed and diagnosed cases [69]. Cheng et al. extrapolated already estimated utility weights for pre-symptomatic hepatitis A to model the preclinical stage of hepatitis E [76]. Assumptions around utility estimates are common, but require careful consideration to avoid a deviation from the initial target population. Although health outcomes are most often captured after treatment begins, some models included screening and diagnostic specific health effects. Risk of perforation due to colonoscopy was included by Atkin et al. in their colorectal cancer CEA [18]. Yang et al. included radiation-induced cancer cases from radiography screening [54]. Failing to include potentially negative health effects of screening tests will overestimate the health benefits and potentially underestimate associated costs.

Some studies reported uncertainty around treatment efficacy inputs [15, 44, 65, 66, 75]. Sensitivity and scenario analyses were broadly used to account for this uncertainty. Not surprisingly, cost-effectiveness estimates were influenced by treatment efficacy of early treatment and uptake [65, 66, 75]. A few studies conducted a value of information analyses to estimate the value of collecting further information to resolve decision uncertainty [18, 44, 75].

### Non-health costs and outcomes, and spillovers

CEAs take into account the costs and outcomes of specific interventions and compare them to determine if they provide enough benefits relative to the cost compared to the next best alternative. However, not all potential benefits and costs are necessarily health related. The perspective of a CEA determines what kind of effects and costs will be included. A healthcare perspective seeks to compare costs and consequences that directly pertain to the healthcare sector. They generally focus on health-related outcomes [81]. Alternatively, a societal perspective attempts to capture all relevant costs and outcomes, health-related or not. Transportation costs, out-of-pocket expenses, and productivity losses are a few examples. These analyses evaluate the trade-off between health and any other outcome, but this information is rarely known, i.e., societal preferences between health and productivity or educational benefits [81]. This review identified 38 (55.8%) and 15 (22%) studies that developed their analyses under a healthcare [12, 14, 18, 20–23, 25, 27, 29, 34–40, 42, 44, 46–55, 58, 60, 62, 65, 66, 68, 69, 75, 78] and societal perspective [13, 15, 17, 19, 28, 33, 41, 43, 56, 57, 59, 61, 67, 76, 80], respectively. The following were specific studies that included non-health costs and/or outcomes: Cressman et al. estimated the productivity loss of lung cancer patients who had been previously working before starting treatment [56]. Phisalprapa et al. included non-medical costs (transportation, meals, accommodations, and facilities) in their evaluation of non-alcoholic fatty liver disease [33]. Pil et al. used a patient questionnaire to assess indirect costs in their skin cancer screening CEA related to productivity loss, morbidity, and early mortality [59]. Sharma et al. included patient transportation costs [61]. The decision to include indirect (or non-medical) costs and outcomes depends on the decision maker’s perspective. The societal perspective allows a thorough analysis by including a broader spectrum of the associated consequences. However, including all indirect outcomes or externalities might prove a difficult task, and missing important outcomes will render the evaluation incomplete and possibly biased. It is also true that although most studies considering a societal perspective focused on costs, there was one that also included non-health benefits or outcomes. Chen et al. compared the benefits of the different types of education that children received after being screened and treated for neonatal hearing loss. Children who were successfully identified and treated for hearing loss were expected to have better educational outcomes [45]. Sensitivity analyses determined that cost-effectiveness estimates were most affected by the inclusion of the societal costs [80].

One concern of adopting a societal perspective is the implicit assumptions on how resources should be distributed; for example, including productivity costs (an important part of non-health outcomes) generally benefits treatments of the working age population at the cost of children and seniors [82]. Prusa et al. developed a CEA of toxoplasmosis screening for children in Austria. Besides considering the projected lifetime productivity loss of the affected children, they also considered the productivity loss of parents [80]. Consequences (health-related or not) that fall on third or external parties are called spillover effects [83]. Spillover effects were not identified or modeled in any other study. Basu and Meltzer argue that CEAs might better reflect all associated costs and outcomes by considering spillovers [83]. CEAs that focus on screening tools have specific challenges to address regarding spillovers or externalities, especially health-related ones. False positive tests for venereal diseases, for instance, can have negative consequences for families and third parties in terms of anxiety, stress, and divorce. On the other hand, there are potential positive spillovers. For example, screening tests might have a modest capacity to identify similar conditions. This review did not identify studies that included benefits of such opportunistic identification.

## Discussion

This study reviewed the latest CEAs of screening tools and provided a thorough breakdown of challenges and suggestions to overcome them. The included studies mentioned several assumptions and methodological alternatives that were grouped in four major categories: the screening pathway, pre-symptomatic disease, treatment outcomes, and spillovers and externalities. To capture all important costs and outcomes of a screening tool, screening pathways should be modeled through the treatment of the patient. Also, false positive and false negative patients are likely to have important costs and benefits and should be included in the analysis. As these patients are difficult to identify in regular data sources, common treatment patterns should be used to determine how these patients are likely to be treated. Many assumptions are needed when modeling screening tools. It is important that these assumptions are clearly indicated and that the consequences of these assumptions are tested in sensitivity analyses. These include the assumptions such as the independence of consecutive tests and the level of patient and provider compliance to guidelines and sojourn times, i.e., the time between when a patient can be identified by screening test and when they would have been identified due to symptoms. As data is rarely available regarding the progression of undiagnosed patients, extrapolation from diagnosed patients may be necessary. Not surprisingly, different scenarios concluded that longer sojourn times were likely to result in improved health outcomes. This becomes one of the main drivers of the effectiveness of a screening test, besides the accuracy at which it identifies patients correctly. This was particularly true when available treatment was capable of modifying disease progression. Finally, non-health costs and outcomes were observed for studies that developed their analyses under a societal perspective. These were not consistently reported, mostly likely due to different guidelines from decision makers.

This review thoroughly examined the latest methodological challenges associated with modeling CEAs of screening tools. However, some limitations are to be noted. Studies focusing on genomic and blood transfusion screening tests were excluded. Genomic screening was excluded because a recent paper evaluated CEAs of genomic screening tests [84]. Blood transfusion tests were excluded because different issues arise when testing blood for treatment rather than testing patients for disease [85]. Challenges and methodologies of CEAs are expected to vary considerably between these groups. Finally, studies were limited to 2017 to capture the most recent state of the art in this area. We were interested in the latest available evidence to appropriately review the most up-to-date methodologies for modeling screening tools from a health economic perspective. However, all diseases were included to avoid disease-specific issues and to provide a broad learning across disease areas.

## Conclusion

Many new screening tools are being developed and require cost-effectiveness analyses to support their value proposition. Screening tools should follow diagnostic guidelines, but have additional challenges given that sojourn times and pre-symptomatic progression data is rarely known. Current cost-effectiveness analyses extrapolate pre-symptomatic progression from symptomatic patients and thoroughly test assumptions in sensitivity analyses, including sojourn times. By following these methodological suggestions, screening tool evaluations are expected to become a better reflection of medical practice and to provide better quality evidence for decision makers making difficult trade-offs between funding screening interventions or other health technologies.

## Appendix 1

**Table 3:** EMBASE search strategy

1	exp mass screening/
2	limit 1 to (human and english language)
3	screen*.mp.
4	limit 3 to (human and english language)
5	exp “cost benefit analysis”/ or exp “cost effectiveness analysis”/ or cost-effective*.mp.
6	limit 5 to (human and english language)
7	2 or 4
8	6 and 7

## Appendix 2

**Table 4:** MEDLINE search strategy

1	exp Mass Screening/
2	limit 1 to (english language and humans)
3	screen*.mp.
4	limit 3 to (english language and humans)
5	exp Cost-Benefit Analysis/ or cost-effective*.mp
6	limit 5 to (english language and humans)
7	economic evaluation.mp.
8	limit 7 to (english language and humans)
9	2 or 4
12	6 or 8
11	9 and 10
